# Role of Aminoglycoside-Modifying Enzymes (AMEs) in Resistance to Aminoglycosides among Clinical Isolates of *Pseudomonas aeruginosa* in the North of Iran

**DOI:** 10.1155/2021/7077344

**Published:** 2021-08-21

**Authors:** Leila Ahmadian, Zahra Norouzi Bazgir, Mohammad Ahanjan, Reza Valadan, Hamid Reza Goli

**Affiliations:** ^1^Molecular and Cell Biology Research Centre, Faculty of Medicine, Mazandaran University of Medical Sciences, Sari, Iran; ^2^Department of Medical Microbiology and Virology, Faculty of Medicine, Mazandaran University of Medical Sciences, Sari, Iran; ^3^Student Research Committee, Faculty of Medicine, Mazandaran University of Medical Sciences, Sari, Iran; ^4^Department of Immunology, Faculty of Medicine, Mazandaran University of Medical Sciences, Sari, Iran

## Abstract

In recent years, the prevalence of resistance to aminoglycosides among clinical isolates of *Pseudomonas aeruginosa* is increasing. The aim of this study was to investigate the role of aminoglycoside-modifying enzymes (AMEs) in resistance to aminoglycosides in clinical isolates of *P. aeruginosa*. The clinical isolates were collected from different hospitals. Disk agar diffusion test was used to determine the antimicrobial resistance pattern of the clinical isolates, and the minimum inhibitory concentration of aminoglycosides was detected by microbroth dilution method. The PCR was performed for discovery of aminoglycoside-modifying enzyme-encoding genes. Among 100 screened isolates, 43 (43%) isolates were resistant to at least one tested aminoglycosides. However, 13 (13%) isolates were resistant to all tested aminoglycosides and 37 isolates were detected as multidrug resistant (MDR). The resistance rates of *P. aeruginosa* isolates against tested antibiotics were as follows: ciprofloxacin (41%), piperacillin-tazobactam (12%), cefepime (32%), piperacillin (26%), and imipenem (31%). However, according to the MIC method, 13%, 32%, 33%, and 37% of the isolates were resistant to amikacin, gentamicin, tobramycin, and netilmicin, respectively. The PCR results showed that *AAC(6*′*)-Ib* was the most commonly (26/43, 60.4%) identified AME-encoding gene followed by *AAC(6*′*)-IIa* (41.86%), *APH(3*′*)-IIb* (34.8%), *ANT(3*^″^*)-Ia* (18.6), *ANT(2*^″^*)-Ia* (13.95%), and *APH(3*^″^*)-Ib* (2.32%). However, *APH(3*′*)-Ib* was not found in any of the studied isolates. The high prevalence of AME-encoding genes among aminoglycoside-resistant *P. aeruginosa* isolates in this area indicated the important role of AMEs in resistance to these antibiotics similar to most studies worldwide. Due to the transmission possibility of these genes between the Gram-negative bacteria, we need to control the prescription of aminoglycosides in hospitals.

## 1. Introduction

*Pseudomonas aeruginosa*, as an opportunist pathogen, is responsible for several nosocomial infections such as bacteremia; urinary tract, blood, respiratory, burn, and soft tissue infections; external otitis; and endocarditis in clinical settings, which are often difficult to treat [1, 2]. Aminoglycosides, fluoroquinolones, and *β*-lactams are clinically effective antibiotics in the treatment of infections caused by *P. aeruginosa*, while carbapenems are the last-line option before colistin [3–5]. Increasing resistance to fluoroquinolones and *β*-lactams has led to attentiveness in clinical applications of aminoglycosides against Gram-negative bacteria [6]. However, *P. aeruginosa* can survive in hospital environments for a long time due to the high-level resistance against biocides and can acquire and/or spread the antibiotic resistance genes [7]. Transfer of these genes, especially in hospital environments, can further complicate the treatment of the infected patients [8]. Some of the most important resistance genes transferable between the Gram-negative bacteria, especially in *P. aeruginosa*, are the aminoglycoside-modifying enzyme- (AME-) encoding genes [6]. Enzymatic modification is one of the important mechanisms leading to resistance against aminoglycosides, while these antibiotics are counting as the most potent drugs of choice for the treatment of life-threatening infections caused by *P. aeruginosa* [8]. The AMEs are classified as acetyltransferases (AACs), nucleotidyltransferases (ANTs), and phosphotransferases (APH) [9]. These enzymes started N-acetylation, O-nucleotidylation, and/or O-phosphorylation of the aminoglycosides resulting in inactivation of the drugs and therapeutic failure [9, 10]. *AAC(6*′*)-I*, *AAC (6*′*)-II*, *ANT(2*^″^*)-I*, and *APH(3*′) are the most common AME-encoding genes in *P. aeruginosa* [11]. The mobile genetic elements such as integrons, plasmids, and transposons can help in the rapid spreading of these clinically important genes [12]. Recently, Thirumalmuthu et al. found that the presence of AME genes was the main cause for resistance to aminoglycosides in multidrug-resistant (MDR) ocular isolates of *P. aeruginosa* [13]. Therefore, understanding the prevalence of these genes is important for the practical control of antibiotic resistance. This study was aimed at evaluating the role of aminoglycoside-modifying enzyme-encoding genes in resistance to these antibiotics in the clinical isolates of *P. aeruginosa* collected from hospitalized patients in the north of Iran.

## 2. Materials and Methods

### 2.1. Sample Collection and Identification of the Bacteria

One hundred consecutive nonduplicated clinical isolates of *P. aeruginosa* were collected during 2018-2019 from 5 different teaching and therapeutic hospitals in the north of Iran. The isolates were obtained from different sources including sputum, urine, wound, catheter, blood, stool, and eye. After identification of the isolates by different conventional microbiological and biochemical methods [14], the pure overnight isolates were cultured in trypticase soy broth (Merck, Germany) containing 10% glycerol and were stocked at -20°C for the next investigations. The isolates which were detected as *P. aeruginosa* were included in the study and others were rejected.

### 2.2. Antibiotic Susceptibility Testing

The antimicrobial susceptibility pattern of the clinical *P. aeruginosa* isolates was determined by the Kirby-Bauer disk agar diffusion method on Mueller–Hinton agar (Merck, Germany), according to the Clinical and Laboratory Standards Institute (CLSI) recommendations against 4 different antibiotics [15]. The following antibiotics were exploited in this study: ciprofloxacin (5 *μ*g), cefepime (30 *μ*g), piperacillin (100 *μ*g), piperacillin/tazobactam (100/10 *μ*g), and imipenem (10 *μ*g) (Rosco, Denmark). However, the minimum inhibitory concentrations (MICs) of amikacin, gentamicin, tobramycin, and netilmicin (Sigma, Germany) were detected using the microbroth dilution method conferring to the CLSI guidelines [15], and the lowest concentration of the antibiotics that inhibited the bacterial growth was reported as the MIC. *P. aeruginosa* ATCC 27853 was used as the control strain in antimicrobial susceptibility testing.

### 2.3. DNA Extraction and Amplification of AME-Encoding Genes

The alkaline lysis method based on sodium dodecyl sulfate (SDS) (Sigma) and NaOH (Sigma) was used for DNA extraction [16]. Briefly, we solved 0.5 g SDS and 0.4 g NaOH in 200 ml distilled water and suspended the bacterial colony in 20 *μ*l of this extraction buffer. Then, the suspension was warmed at 95°C for 10 min and centrifuged in13000 × gfor 3 min. Next, 180 *μ*l distilled water was added to it and saved at -20°C as extracted DNA until use. All isolates which were resistant to at least one aminoglycoside were examined by the polymerase chain reaction (PCR) method, and the presence of aminoglycoside-modifying enzyme-encoding genes was detected using specific primers (Table 1). The detection of *ANT(3*^″^*)-Ia*, *ANT (2*^″^*)-Ia*, *APH(3*′*)-IIb*, *AAC(6*′*)-Ib*, *APH (3*′*)-Ib*, *APH (3*^″^*)-Ib*, and *AAC (6*′*)-IIa* genes was performed by the PCR method. The PCRs were done in a final volume of 15 *μ*l reaction mixtures containing 7.5 *μ*l of Master Mix (Ampliqon, Denmark), 300 ng of the extracted DNA, and 10 pmol of each primer and added distilled water to the final volume. The thermal cycler (Bio-Rad, USA) running condition was as follows: an initial denaturation for 5 min at 94°C and 35 cycles including denaturation at 94°C for 60 seconds, annealing for 1 min at specific primer set temperatures (Table 1), and extension at 72°C for 2 min, and after the cycles, a final extension of the amplicons happened at 72°C for 10 min. Finally, the amplified DNAs were visualized by electrophoresis on 1% agarose gel (Sigma, Germany) containing a 3% safe stain (SinaClon, Iran). Distilled water was used as the negative control in PCR.

### 2.4. Ethical Approval Statements

We received the clinical samples without names from the laboratories of the hospitals affiliated to the Mazandaran University of Medical Sciences. This study was conducted in accordance with the Declaration of Helsinki; however, written informed consent form was provided by the patients or a close relative before hospitalization, and classifying information of each sample was kept secret. Also, this study was approved by the Iran National Committee for Ethics in Biomedical Research with the national ethical code (consent ref number) IR.MAZUMS.REC.1398.980.

## 3. Results

### 3.1. Patients and Samples

A total of 100 *P. aeruginosa* clinical isolates, collected from 60 males and 40 females with an average age of 46 years, were analyzed in the present study. The isolates were collected from five educational-therapeutic hospitals including Zare (burn center, 11 isolates), Razi (infectious center, 22 isolates), Bu-Ali Sina (pediatric center, 17 isolates), Fatemeh Zahra (heart center, 10 isolates), and Imam Khomeini (general center, 40 isolates). However, the isolates were collected from different hospital wards counting intensive care unit (ICU) (48 isolates), burn (9 isolates), internal medicine (4 isolates), operation room (3 isolates), men (3 isolates), women (2 isolates), emergency (13 isolates), surgery (3 isolates), oncology (1 isolate), cardiac care unit (CCU) (6 isolates), neurology (2 isolates), and pediatric (6 isolates). In terms of sample type, the isolates were obtained from sputum (*n* = 37), urine (*n* = 26), wound (*n* = 20), catheter (*n* = 8), blood (*n* = 5), stool (*n* = 2), and eye (*n* = 2).

### 3.2. Antimicrobial Susceptibility Pattern of the Isolates

**Figure 1:**
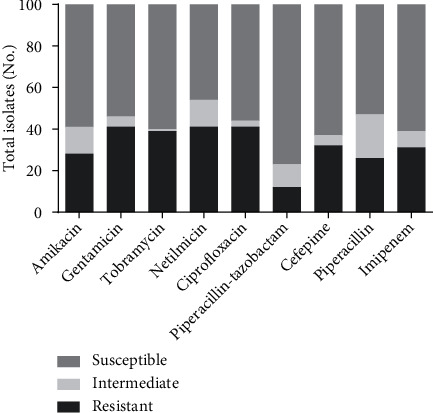
Antibiotic resistance pattern of 100 *Pseudomonas aeruginosa* clinical isolates.

The resistance rates against all tested antibiotics are shown in Figure 1. According to the antimicrobial susceptibility testing, 43 (43%) isolates were resistant to at least one tested aminoglycoside. However, 13 (13%) isolates were resistant to all tested aminoglycosides in the present study. The highest aminoglycoside resistance rate was observed against gentamicin (*n* = 41), while 39 and 28 isolates were resistant towards tobramycin and amikacin, respectively. Piperacillin-tazobactam was the most effective antibiotic in this study, while the highest resistance rate was shown against gentamicin and ciprofloxacin. Also, 37 isolates were detected as MDR in the present study.

The microbroth dilution results indicated that 6, 23, 12, and 2 isolates were high-level resistant (MIC ≥ 512 *μ*g/ml) to amikacin, gentamicin, tobramycin, and netilmicin, respectively (Table 2).

### 3.3. Genotypic Detection of AME-Encoding Genes

The PCR amplification exhibited that 34/43 (79%) of aminoglycoside-resistant isolates were positive for AME-encoding genes (Table 3). *AAC(6*′*)-Ib* was the most commonly (26/43, 60.4%) identified gene followed by *AAC(6*′*)-IIa* (41.86%), *APH(3*′*)-IIb* (34.8%), *ANT(3*^″^*)-Ia* (18.6%), *ANT(2*^″^*)-Ia* (13.95%), and *APH(3*^″^*)-Ib* (2.32%). *APH(3*′*)-Ib* was not found in any of the studied isolates. Among the 34 isolates that contained at least one of the studied genes, 12 (35.29%) isolates were carrying only one AME-encoding gene; however, 12 (35.29%) isolates were positive for two genes, 4 (11.76%) isolates had 3 genes, 4 (11.76%) others contained 4 AME-encoding genes, and 2 (5.88%) isolates had 5 AME genes. We did not detect any isolates containing all studied genes in the present study (Table 3). The simultaneous presence of the genes encoding aminoglycoside-modifying enzymes had a significant effect on the increase of the MICs of gentamicin and tobramycin (*p* < 0.05), whereas the combination of the two genes had less effect on the increasing of amikacin and netilmicin MICs. Interestingly, among 26 ICU isolates in this study, 22 (84.61%) of them have contained at least one AME-encoding gene, while this rate among burn isolates was 6/8 (75%). Also, Table 4 exhibits the relation between the aminoglycoside resistance phenotype and the simultaneous presence of AME-encoding genes in the studied isolates. According to this table, most aminoglycoside resistance phenotype (29/43, 67.44%) was the simultaneous resistance against gentamicin and tobramycin. However, different resistance phenotypes in the present study showed similar resistance gene profile.

## 4. Discussion

The aminoglycosides as the broad-spectrum antibiotics have remained useful as antipseudomonal choice agents for the treatment of life-threatening infections [17]. Therefore, the continuous increase of aminoglycoside resistance levels among the clinical isolates of Gram-negative bacteria will become a growing clinical concern in the future [17, 18]. The lowest resistance to aminoglycosides in the present study was seen against amikacin (13%) with the MICs ranging from 0.25 to 2048 *μ*g/ml, whereas 37% of the *P. aeruginosa* isolates were resistant to netilmicin with the MICs ranging from 4 *μ*g/ml to 2048 *μ*g/ml. Moreover, Kashfi et al. reported that amikacin was more effective than gentamicin against *P. aeruginosa* isolated from burned patients [19], while their MICs ranged from 2 *μ*g/ml to 256 *μ*g/ml. These lowest resistances against amikacin may be due to the lower prescription of this aminoglycoside in hospitals of Iran. On the other hand, the rate of resistance to aminoglycosides is different in various regions and countries, even in different hospitals of the same region in a similar country. These variabilities may be due to the different causes such as overuse of these drugs in hospitals, arbitrary use of the drugs by people without a prescription, geographical and cultural differences, countries' health levels, and hygienic condition [20].

Aminoglycoside resistance in *P. aeruginosa* is often related to the production of various aminoglycoside-modifying enzymes [19], so the more prevalence of these enzymes is an important problem. In this study, the aminoglycoside resistance rate was 43%, while 79% of the resistant isolates carried AME-encoding genes. In total, 34 AME patterns (28 combinations and 6 single-gene forms) were identified, which showed different levels of aminoglycoside resistance. As an important result of the present study, we found that the simultaneous presence of AME genes was the cause of increasing the MIC ranges of gentamicin and tobramycin. This shows that these genes were the most effective factor in resistance to tested aminoglycosides, while this effect was lower about amikacin and netilmicin. According to the study conducted by Panahi et al. in 2020, AMEs were highly prevalent (96.2%) among the aminoglycoside nonsusceptible *P. aeruginosa* isolates [21]. However, Odumosu et al. in Nigeria reported that the AME-encoding genes *AAC(6*′*)–I* and *ANT(2*^″^*)–I* were found only in 22.22% of their isolates [8].

In the present study, *AAC(6*′*)-Ib*, *AAC(6*′*)-IIa*, and *APH(3*′*)-IIb* were more prevalent than the *ANT(3*^″^*)-Ia*, *ANT(2*^″^*)-Ia*, and *APH(3*^″^*)-Ib* genes among *P. aeruginosa* clinical isolates. Also, *APH(3*′*)-Ib* was not found in any of the isolates. The more prevalence of *AAC(6*′*)-Ib* in our study was similar to the previous reports from Iran [22, 23], while it was significantly higher compared with another research conducted by Vaziri et al. in Iran which reported a 7% prevalence of this gene [17]. Our results confirmed that the presence of *AAC(6*′*)-Ib* gene may be more effective in resistance of *P. aeruginosa* towards tested aminoglycosides, although probably other mechanisms contribute to this type of resistance, too [24]. However, the high prevalence of this gene in *P. aeruginosa* clinical isolates collected from Iranian patients demonstrates its key role in resistance to aminoglycosides and its high distribution in Iran [21–23]. While the high prevalence of the *AAC(6*′*)-I* gene can result in a higher resistance level to amikacin [10], our MIC results showed that 42.3% of *AAC(6*′*)-Ib*-positive isolates were resistant to this antibiotic.

The frequency of *AAC(6*′*)-IIa* in similar studies was 1.9% in France [25], 18.5% in Nigeria [8], and 10% in another study carried out in Iran [19]. However, 18% of our all clinical isolates were *AAC(6*′*)-IIa* positive, indicating the increasing frequency of this gene in our region. Furthermore, *AAC(6*′*)-IIa* was known as the most prevalent aminoglycoside resistance gene in Europe [26]. On the other hand, this gene can spread by integrons, causing the high prevalence of this resistance gene which plays an effective role in resistance to aminoglycosides [27]. *AAC(6*′*)-II* has a key role in resistance towards gentamicin, tobramycin, netilmicin, and kanamycin [27]. However, among the *AAC(6*′*)-IIa*-positive isolates of this study, 83%, 88%, and 77% of the isolates were resistant to gentamicin, tobramycin, and netilmicin, respectively.

It seems that *ANT(2*^″^*)-Ia* has a relatively important role in resistance against gentamicin and tobramycin in *P. aeruginosa* isolates [28]. Interestingly, all *ANT(2*^″^*)-Ia*-positive isolates in the present study were resistant to these aminoglycosides. However, 13.9% of our isolates were positive for this gene that was comparable with another study conducted in Iran [22]. On the other hand, other studies conducted in Iran and South Korea reported the *ANT(2*^″^*)-Ia* gene as the most common aminoglycoside resistance gene [19, 29]. Moreover, Michalska et al. in Poland and Odumosu et al. in Nigeria detected 36% and 16.6% of this gene in their *P. aeruginosa* clinical isolates, respectively [8, 30].

The results of our study revealed that 18.6% of the *P. aeruginosa* clinical isolates were carrying *ANT(3*^″^*)-Ia* gene similar to the study of Aghazadeh et al. in Iran [22], while 87.5% of other research in Tehran contained this gene [19]. This significant difference may be due to the different sources of the isolates, as 38% of the bacteria in the first study were isolated from the sputum of the cystic fibrosis patients and 62% of their isolates were collected from burned patients [22], while all isolates in another mentioned study [19] were collected from burned patients. Generally, burn patients are at high risk for bacterial infections, so isolates collected from burn injuries may show a higher resistance level [31].

Although *APH(3*^″^*)-Ib* is responsible for streptomycin resistance in *Enterobacteriaceae* [10], 2.32% of our isolates were positive for this gene. This was comparable to Michalska et al.'s study in Poland from which 8% of their isolates were carrying this gene [30]. On the other hand, besides the fact that we found that 34.8% of our isolates were *APH(3*′*)-IIb* positive, this was lower than the similar studies carried out in Iran and Thailand with the rate of 61.8% and 57%, respectively [22, 32]. Besides, we detected any isolates with the presence of the *APH(3*′*)-Ib* gene, while in other studies conducted in Iran, the frequency of this gene was 60% and 46%, respectively [19, 22].

## 5. Conclusions

The aminoglycoside-modifying enzyme-encoding genes are highly prevalent among *Pseudomonas aeruginosa* clinical isolates worldwide, especially in Iran. The inappropriate and indiscriminate prescription of aminoglycosides was probably one of the main reasons for the high prevalence of some aminoglycoside resistance genes in this study. Due to the high ability of *P. aeruginosa* in the distribution of these genes, an appropriate antibiotic stewardship policy is required for the prevention of AME gene spreading and to decrease the aminoglycoside resistance rates.

## Figures and Tables

**Table 1:** Primers used for detection of aminoglycoside-modifying enzyme-encoding genes by PCR.

Target genes	Primer sequences (5′ to 3′)	Amplicon size (bp)	Annealing temperature (°C)	References
*ANT(3* ^″^ *)-Ia*	Pri F: TGT AGA AGT CAC CAT TGT TG	152	51	This study
	Pri R: TCA GCA AGA TAG CCA GAT			
*ANT(2* ^″^ *)-Ia*	Pri F: GCA GGT CAC ATT GAT ACA C	225	54	This study
	Pri R: TCC GCT AAG AAT CCA TAG TC			
*AAC(6*′*)-Ib*	Pri F: GAC CAA CAG CAA CGA TTC	375	57	This study
	Pri R: AAC AGC AAC TCA ACC AGA			
*AAC(6*′*)-IIa*	Pri F: CCA TAA CTC TTC GCC TCA T	442	48	This study
	Pri R: AAT CCT GCC TTC TCA TAG C			
*APH(3*′*)-IIb*	Pri F: TTC GTC AAG CAG GAA GTC	662	50	This study
	Pri R: TAG AAG AAC TCG TCC AAT AGC			
*APH(3*′*)-Ib*	Pri F: TTG TTG TTG ACG CAT TGG	284	56	This study
	Pri R: GCC GAC TAC CTT ACC TTC			
*APH(3* ^″^ *)-Ib*	Pri F: GGT GAT AAC GGC AAT TCC	548	56	This study
	Pri R: GGT CCA ATC GCA GAT AGA			

**Table 2:** The aminoglycoside MICs against 43 aminoglycoside-resistant *Pseudomonas aeruginosa*.

MIC ranges (*μ*g/ml)	Amikacin				Gentamicin				Tobramycin				Netilmicin			
	≤16	32	64-128	≥256	≤4	8	16-128	≥256	≤4	8	16-128	≥256	≤8	16	32-128	≥256
No. of isolates	25	5	7	6	10	1	6	26	9	1	17	16	4	2	30	7

**Table 3:** The correlation between the AME gene profile of *P. aeruginosa* clinical isolates and the MIC ranges of aminoglycosides.

Genotypes	No. (%) of MDRs	No. (%) of isolates with different MICs (*μ*g/ml) against amikacin				No. (%) of isolates with different MICs (*μ*g/ml) against gentamicin				No. (%) of isolates with different MICs (*μ*g/ml) against tobramycin				No. (%) of isolates with different MICs (*μ*g/ml) against netilmicin			
		≤16	32	64-128	≥256	≤4	8	16-128	≥256	≤4	8	16-128	≥256	≤8	16	32-128	≥256
*ANT(3*^″^*)-Ia* (*n* = 8)	7 (87.5)	5 (62.5)	1 (12.5)	0	2 (25)	1 (12.5)	0	2 (25)	5 (62.5)	1 (12.5)	0	6 (75)	1 (12.5)	1 (12.5)	0 (0)	7 (87.5)	0
*ANT(2*^″^*)-Ia* (*n* = 6)	5 (83.3)	2 (33.3)	2 (33.3)	2 (33.3)	0	0	0	2 (33.3)	4 (66.6)	0	0	4 (66.6)	2 (33.3)	1 (16.6)	1 (16.6)	3 (50)	1 (16.6)
*AAC(6*′*)-Ib* (*n* = 26)	24 (92.3)	11 (42.3)	4 (15.3)	7 (26.9)	4 (15.3)	1 (3.8)	1 (3.8)	3 (11.5)	21 (80.7)	1 (3.8)	0	12 (46.1)	13 (50)	3 (11.5)	0	18 (69.2)	5 (19.2)
*AAC(6*′*)-IIa* (*n* = 18)	16 (88.8)	10 (55.5)	3 (16.6)	3 (16.6)	2 (11.1)	2 (11.1)	1 (5.5)	4 (22.2)	11 (61.1)	2 (11.1)	0	10 (55.5)	6 (33.3)	4 (22.2)	0	12 (66.6)	2 (11.1)
*APH(3*′*)-IIb* (*n* = 15)	13 (86.6)	7 (46.6)	3 (20)	3 (20)	2 (13.3)	1 (6.6)	0	4 (26.6)	10 (66.6)	1 (6.6)	0	9 (60)	5 (33.3)	2 (13.3)	1 (6.6)	10 (66.6)	2 (13.3)
*APH(3*^″^*)-Ib* (*n* = 1)	0	1 (100)	0	0	0	0	0	1 (100)	0	0	0	1 (100)	0	0	1 (100)	0	0
*ANT(3*^″^*)-Ia+ ANT(2*^″^*)Ia* (*n* = 2)	2 (100)	1 (50)	1 (50)	0	0	0	0	1 (50)	1 (50)	0	0	1 (50)	1 (50)	1 (50)	0	1 (50)	0
*ANT(3*^″^*)-Ia+ AAC(6*′*)-Ib* (*n* = 6)	5 (83.3)	4 (66.6)	1 (16.6)	0	1 (16.6)	0	0	2 (33.3)	4 (66.6)	0	0	5 (83.3)	1 (16.6)	1 (16.6)	0	5 (83.3)	0
*ANT(3*^″^*)-Ia+ AAC(6*′*)-IIa* (*n* = 3)	2 (66.6)	2 (66.6)	1 (33.3)	0	0	0	0	1 (33.3)	2 (66.6)	0	0	2 (66.6)	1 (33.3)	1 (33.3)	0	2 (66.6)	0
*ANT(3*^″^*)-Ia+ APH(3*′*)-IIb* (*n* = 4)	3 (75)	2 (50)	1 (25)	0	1 (25)	0	0	1 (25)	3 (75)	0	0	3 (75)	1 (25)	1 (25)	0	3 (75)	0
*ANT(2*^″^*)-Ia+ AAC(6*′*)-Ib* (*n* = 5)	5 (100)	1 (20)	2 (40)	2 (40)	0	0	0	1 (20)	4 (80)	0	0	2 (40)	3 (60)	1 (20)	0	3 (60)	1 (20)
*ANT(2*^″^*)-Ia+ AAC(6*′*)-IIa* (*n* = 5)	5 (100)	1 (20)	2 (40)	2 (40)	0	0	0	1 (20)	4 (80)	0	0	2 (40)	3 (60)	1 (20)	0	3 (60)	1 (20)
*ANT(2*^″^*)-Ia+ APH(3*′*)-IIb* (*n* = 6)	5 (83.3)	2 (33.3)	2 (33.3)	2 (33.3)	0	0	0	2 (33.3)	4 (66.6)	0	0	3 (50)	3 (50)	1 (16.6)	1 (16.6)	3 (50)	1 (16.6)
*ANT(2*^″^*)-Ia+ APH(3*^″^*)-Ib* (*n* = 1)	0	1 (100)	0	0	0	0	0	1 (100)	0	0	0	1 (100)	0	0	1 (100)	0	0
*AAC(6*′*)-Ib+ AAC(6*′*)-IIa* (*n* = 14)	12 (85.7)	7 (50)	2 (14.2)	3 (21.4)	2 (14.2)	0	1 (7.1)	2 (14.2)	11 (78.5)	0	0	8 (57.1)	6 (42.8)	3 (21.4)	0	9 (64.2)	2 (14.2)
*AAC(6*′*)-Ib+ APH(3*′*)-IIb* (*n* = 9)	8 (88.8)	3 (33.3)	2 (22.2)	3 (33.3)	1 (11.1)	0	0	1 (11.1)	8 (88.8)	0	0	5 (55.5)	4 (44.4)	1 (11.1)	0	6 (66.6)	2 (22.2)
*AAC(6*′*)-IIa+ APH(3*′*)-IIb* (*n* = 12)	11 (91.66)	5 (41.6)	3 (25)	3 (25)	1 (8.3)	1 (8.3)	0	3 (25)	8 (66.6)	1 (8.3)	0	7 (58.3)	4 (33.3)	2 (16.6)	0	8 (66.6)	2 (16.6)
*APH(3*′*)-IIb+ APH(3*′′*)-Ib* (*n* = 1)	0	1 (100)	0	0	0	0	0	1 (100)	0	0	0	1 (100)	0	0	1 (100)	0	0
*ANT(3*^″^*)-Ia*+ *ANT(2*^″^*)-Ia+ AAC(6*′*)-Ib* (*n* = 2)	2 (100)	1 (50)	1 (50)	0	0	0	0	1 (50)	1 (50)	0	0	1 (50)	1 (50)	1 (50)	0	1 (50)	0
*ANT(3*^″^*)-Ia*+ *ANT(2*^″^*)-Ia+ AAC(6*′*)-IIa* (*n* = 2)	2 (100)	1 (50)	1 (50)	0	0	0	0	1 (50)	1 (50)	0	0	1 (50)	1 (50)	1 (50)	0	1 (50)	0
*ANT(3*^″^*)-Ia*+ *ANT(2*^″^*)-Ia+ APH(3)-IIb* (*n* = 2)	2 (100)	1 (50)	1 (50)	0	0	0	0	1 (50)	1 (50)	0	0	1 (50)	1 (50)	1 (50)	0	1 (50)	0
*ANT(3*^″^*)-Ia+ APH(3)-IIb+ AAC(6*′*)-Ib* (*n* = 3)	2 (66.6)	2 (66.6)	1 (33.3)	0	0	0	0	1 (33.3)	2 (66.6)	0	0	0	0	1 (33.3)	0	2 (66.6)	0
*ANT(3*^″^*)-Ia+ APH(3*′*)-IIb+ AAC(6*′*)-IIa* (*n* = 3)	2 (66.6)	2 (66.6)	1 (33.3)	0	0	0	0	1 (33.3)	2 (66.6)	0	0	2 (66.6)	1 (33.3)	1 (33.3)	0	2 (66.6)	0
*ANT(3*^″^*)-Ia+ AAC(6*′*)-Ib+ AAC(6*′*)-IIa* (*n* = 3)	2 (66.6)	2 (66.6)	1 (33.3)	0	0	0	0	1 (33.3)	2 (66.6)	0	0	2 (66.6)	1 (33.3)	1 (33.3)	0	2 (66.6)	0
*ANT(2*^″^*)-Ia+ APH(3*′*)-IIb+ AAC(6*′*)-Ib* (*n* = 5)	5 (100)	1 (20)	2 (40)	2 (40)	0	0	0	1 (20)	4 (80)	0	0	3 (60)	2 (40)	1 (20)	0	3 (60)	1 (20)
*ANT(2*^″^*)-Ia+ AAC(6*′*)-Ib+ AAC(6*′*)-IIa* (*n* = 5)	5 (100)	1 (20)	2 (40)	2 (40)	0	0	0	1 (20)	4 (80)	0	0	3 (60)	2 (40)	1 (20)	0	3 (60)	1 (20)
*APH(3*′*)-IIb+ AAC(6*′*)-Ib+ AAC(6*′*)-IIa* (*n* = 9)	8 (88.8)	3 (33.3)	2 (22.2)	3 (33.3)	1 (11.1)	0	0	1 (11.1)	8 (88.8)	0	0	5 (55.5)	4 (44.4)	1 (11.1)	0	6 (66.6)	2 (22.2)
*ANT(2*^″^*)-Ia+ APH(3*′*)-IIb+ APH(3*^″^*)-Ib* (*n* = 1)	0	1 (100)	0	0	0	0	0	1 (100)	0	0	0	1 (100)	0	0	1 (100)	0	0
*ANT(3*^″^*)-Ia+ ANT(2*^″^*)-Ia+ APH(3*′*)-IIb+ AAC(6*′*)-Ib* (*n* = 2)	2 (100)	1 (50)	1 (50)	0	0	0	0	1 (50)	1 (50)	0	0	1 (50)	1 (50)	1 (50)	0	1 (50)	0
*ANT(2*^″^*)-Ia+ APH(3*′*)-IIb+ AAC(6*′*)-Ib+ AAC(6*′*)-IIa* (*n* = 5)	5 (100)	1 (20)	2 (40)	2 (40)	0	0	0	1 (20)	4 (80)	0	0	3 (60)	2 (40)	1 (20)	0	3 (60)	1 (20)
*ANT(3*^″^*)-Ia+ APH(3*′*)-IIb+ AAC(6*′*)-Ib+ AAC(6*′*)-IIa* (*n* = 3)	2 (66.6)	2 (66.6)	1 (33.3)	0	0	0	0	1 (33.3)	2 (66.6)	0	0	2 (66.6)	1 (33.3)	1 (33.3)	0	2 (66.6)	0
*ANT(3*^″^*)-Ia+ ANT(2*^″^*)-Ia+ AAC(6*′*)-Ib+ AAC(6*′*)-IIa* (*n* = 2)	2 (100)	1 (50)	1 (50)	0	0	0	0	1 (50)	1 (50)	0	0	1 (50)	1 (50)	1 (50)	0	1 (50)	0
*ANT(3*^″^*)-Ia+ ANT(2*^″^*)-Ia+ APH(3*′*)-IIb+ AAC(6*′*)-IIa* (*n* = 2)	2 (100)	1 (50)	1 (50)	0	0	0	0	1 (50)	1 (50)	0	0	1 (50)	1 (50)	1 (50)	0	1 (50)	0
*ANT(3*^″^*)-Ia+ ANT(2*^″^*)-Ia+ APH(3*′*)-IIb+ AAC(6*′*)-Ib+ AAC(6*′*)-IIa* (*n* = 2)	2 (100)	1 (50)	1 (50)	0	0	0	0	1 (50)	1 (50)	0	0	1 (50)	1 (50)	1 (50)	0	1 (50)	0

**Table 4:** Relation between the aminoglycoside resistance phenotypes and the presence of AME genes.

Resistance phenotypes	AME-encoding gene profiles	No. of isolates
GM	*AAC(6*′*)-Ib + AAC(6*′*)-IIa*	1
NM	*AAC(6*′*)-IIa + ANT(3*^″^*)-Ia*	2
AK+GM	*ANT(3*^″^*)-Ia + ANT(2*^″^*)-Ia + APH(3*′*)-IIb + AAC(6*′*)-Ib + AAC(6*′*)-IIa*	12
AK+TOB	*ANT(3*^″^*)-Ia + ANT(2*^″^*)-Ia + APH(3*′*)-IIb + AAC(6*′*)-Ib + AAC(6*′*)-IIa*	12
AK+NM	*ANT(3*^″^*)-Ia + ANT(2*^″^*)-Ia + APH(3*′*)-IIb + AAC(6*′*)-Ib + AAC(6*′*)-IIa*	12
GM+TOB	*ANT(3*^″^*)-Ia + ANT(2*^″^*)-Ia + APH(3*′*)-IIb + AAC(6*′*)-Ib + AAC(6*′*)-IIa + APH(3*^″^*)-Ib*	29
GM+NM	*ANT(3*^″^*)-Ia + ANT(2*^″^*)-Ia + APH(3*′*)-IIb + AAC(6*′*)-Ib + AAC(6*′*)-IIa + APH(3*^″^*)-Ib*	27
TOB+NM	*ANT(3*^″^*)-Ia + ANT(2*^″^*)-Ia + APH(3*′*)-IIb + AAC(6*′*)-Ib + AAC(6*′*)-IIa + APH(3*^″^*)-Ib*	27
AK+GM+TOB	*ANT(3*^″^*)-Ia + ANT(2*^″^*)-Ia + APH(3*′*)-IIb + AAC(6*′*)-Ib + AAC(6*′*)-IIa*	12
AK+GM+NM	*ANT(3*^″^*)-Ia + ANT(2*^″^*)-Ia + APH(3*′*)-IIb + AAC(6*′*)-Ib + AAC(6*′*)-IIa*	12
GM+TOB+NM	*ANT(3*^″^*)-Ia + ANT(2*^″^*)-Ia + APH(3*′*)-IIb + AAC(6*′*)-Ib + AAC(6*′*)-IIa + APH(3*^″^*)-Ib*	27

Abbreviations: GM: gentamicin; NM: netilmicin; AK: amikacin; TOB: tobramycin.

## Data Availability

All data generated or analyzed during this study are included in this published article.
